# Autoimmune GFAP astrocytopathy after viral encephalitis: a case report of bimodal overlapping encephalitis

**DOI:** 10.3389/fimmu.2023.1258048

**Published:** 2023-09-13

**Authors:** Ping Cheng, Wenjuan Huang, Meifang Yang, Zhiren Chen, Yifan Geng, Xia Zhang, Weiwei Chen

**Affiliations:** ^1^ Department of Neurology, Graduate School, Bengbu Medical College, Bengbu, Anhui, China; ^2^ Department of Neurology, Xuzhou Central Hospital, XuZhou Clinical School of Xuzhou Medical University, Xuzhou, Jiangsu, China; ^3^ Department of Neurology, Xuzhou Clinical College, Xuzhou Medical University, Xuzhou, Jiangsu, China

**Keywords:** glial fibrillary acidic protein (GFAP), viral encephalitis (VE), herpes simplex viral encephalitis (HSVE), encephalitis, case report

## Abstract

Autoimmune glial fibrillary acidic protein (GFAP) astrocytopathy is a treatable autoimmune disorder affecting the central nervous system. Despite extensive research, the exact etiology and pathogenesis of this condition remain unclear. In recent years, autoimmune encephalitis (AE) after viral encephalitis (VE) has gathered significant attention. Here, we present a case report of autoimmune GFAP astrocytopathy after VE in a 43-year-old Asian male with a history of oral and labial herpes. The patient presented with high-grade fever, headache, urinary retention, unresponsiveness, and apathy. Elevated levels of protein and GFAP-IgG were observed in the cerebrospinal fluid (CSF), and enhanced brain magnetic resonance imaging (MRI) revealed linear enhancement oriented radially to the ventricles. Treatment with intravenous immunoglobulin (IVIG) resulted in symptom relief, reduced lesion enhancement, and decreased protein levels. This case report highlights bimodal encephalitis with no discernible interval between VE and autoimmune GFAP astrocytopathy, which poses diagnostic challenges. Notably, autoimmune GFAP astrocytopathy is a novel form of autoimmune encephalitis, and its treatment lacks sufficient clinical experience. Intriguingly, our patient demonstrated sensitivity to IVIG, a treatment that differed from past reports. Therefore, further exploration of treatment strategies for this condition is warranted.

## Introduction

Autoimmune encephalitis was initially described in 2007 as a form of central nervous system injury mediated by autoimmune mechanisms. The clinical manifestations of this condition include cognitive impairment, mental and behavioral abnormalities, neurological deficits, and seizures of varying degrees. Detecting antibodies associated with autoimmune encephalitis in cerebrospinal fluid or serum can aid in the diagnosis, and immunotherapy has proven effective. VE is defined as inflammation of the brain parenchyma caused by viral infection, often manifested by fever, headache, convulsions, impaired consciousness, meningeal irritation, or neurologic signs. Herpes simplex viral encephalitis (HSVE) is the most common form of viral encephalitis. HSVE typically follows a monophasic course, but relapses have been reported, with in-depth clinical research, it was found that new neurological signs or symptoms appear during recovery or existing symptoms worsen (1). Patients may test negative for HSV virus nucleic acid in cerebrospinal fluid and serum during this period, and antiviral drug therapy is ineffective. However, symptoms improve following immunotherapy, suggesting the occurrence of concomitant autoimmune encephalitis. Since the discovery of autoimmune encephalitis, a new approach to diagnosis and treatment has been proposed for the recurrence after partial remission of HSVE. Here, we report a case of virus encephalitis complicated by autoimmune encephalitis. After early antiviral treatment, the symptoms were not completely relieved and subsequently worsened, resulting in cognitive impairment, mental and behavioral abnormalities, and neurological symptoms. The presence of positive anti-GFAP antibodies in both serum and cerebrospinal fluid, coupled with characteristic brain MRI findings, led to a diagnosis of autoimmune encephalitis, and immunotherapy resulted in clinical and imaging improvement. Autoimmune encephalitis induced by VE is the most common type. In the clinical diagnosis and treatment of suspected cases, searching for these antibodies in serum and cerebrospinal fluid can aid in early diagnosis for patients with poor treatment outcomes.

## Case description

A 43-year-old Asian male suffered from dizziness and headache, with the highest temperature of 39°C. Brain CT scans did not show abnormalities, and initial anti-infective treatment had poor efficacy. The patient reported the gradual onset of vomiting accompanied by intractable hiccups. Three days later, he experienced difficulty with urination and urinary retention, requiring catheterization. CT scans chest abdomen did not show any lesions. He also began to experience limb weakness, hyperhidrosis, and mild cognitive impairment. Within a few days, the patient’s symptoms progressed to include mental and behavioral abnormalities and difficulty walking. As a result of his worsening condition, the patient was transferred to our hospital for further evaluation and treatment.

**Figure 1:**
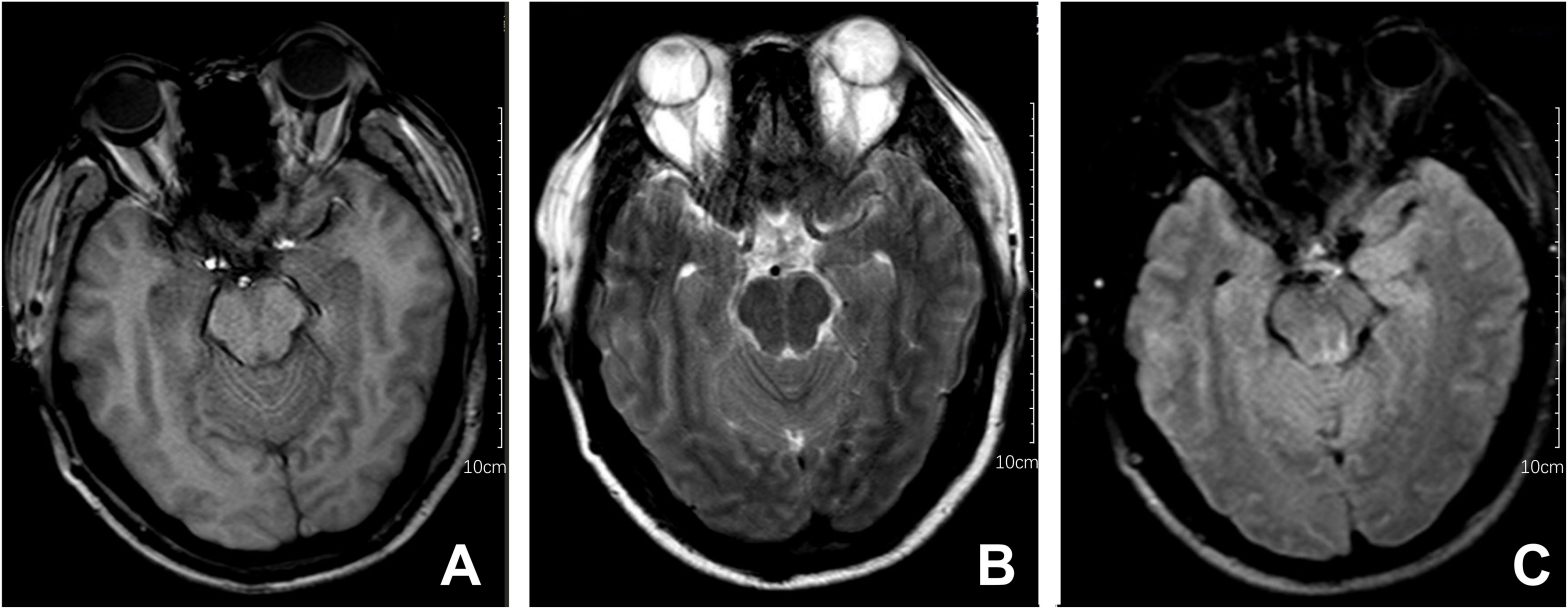
Brain MRI series of the patient. The following findings are described in the figure panel: **(A)** No abnormal signal is found on the T1 sequence; **(B)** Brain MRI revealed T2 hyperintense lesions in the cerebral cortex and bilateral hippocampus; **(C)** A corresponding fluid-attenuated inversion recovery (FLAIR) sequence shows extensive hyperintense signals in the cerebral cortex and bilateral hippocampus.

Upon admission, the patient exhibited cognitive decline, including disorientation in time and location, and could not cooperate with memory and calculation tests. Additionally, the meningeal irritation sign was positive. The patient has a high fever, and to clarify the cause, we got a chest CT, which showed no abnormalities. Serum viral assay revealed increased levels of IgG against herpes simplex virus type 1 (HSV-1) and cytomegalovirus (CMV). A brain MRI showed diffuse and extensive T2/FLAIR hypersignal in the cerebral cortex and bilateral hippocampus (**Figure 1**). Lumbar puncture examination revealed an opening pressure of 210 mmHg, protein content of 1.220 g/L, cerebrospinal fluid white cell count increased to 310*10^6^/L (98% monocytes), and glucose of 2.67 mmol/L. Metagenomic next-generation sequencing of viral and bacterial genomes from the CSF was normal. Based on these findings, the patient was initially diagnosed with viral encephalitis and was treated with intravenous acyclovir administered three times a day (**Table 1**).

**Table 1:** CSF characteristics before and after treatment.

	Lumbar puncture 1	Lumbar puncture 2	Lumbar puncture 3	Lumbar puncture 4
WBC (*10^6^/L)	310	304	45	11
Monocytes (%)	98	95	99	95
Coenocyte (%)	2	5	1	5
Protein (g/L)	1.220	1.240	0.580	0.340
Glucose (mmol/L)	2.67	3.11	2.86	3.45
CSFP (mmH_2_O)	210	150	110	135

CSFP, Cerebrospinal fluid pressure.

**Figure 2:**
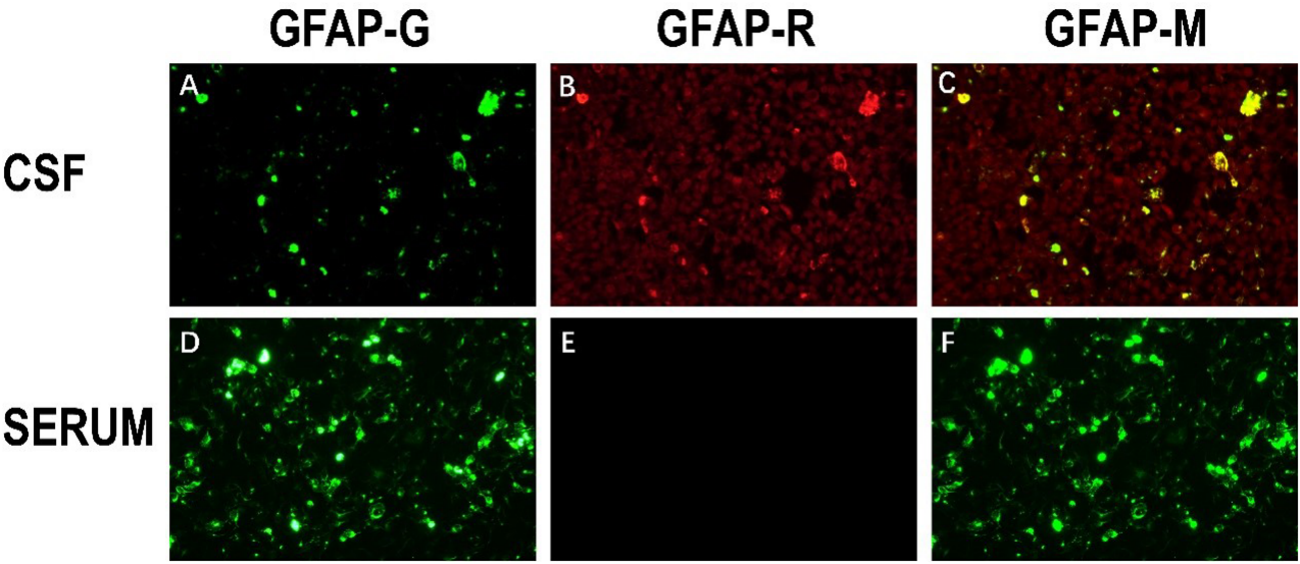
The immunoreactivity of patient’s GFAP-IgG via cell-based assay (CBA) in CSF and serum. **(A)** HEK293 cells stably express green fluorescent proteintagged GFAPa. **(B)** The red fluorescence represents the GFAP antibody in the sample; **(C)** Colocalization of the patient’s GFAP IgG, GFAPa is yellow in the merged images **(A, B)**; **(D–F)** Negative serum test results of anti-GFAP antibody.

**Figure 3:**
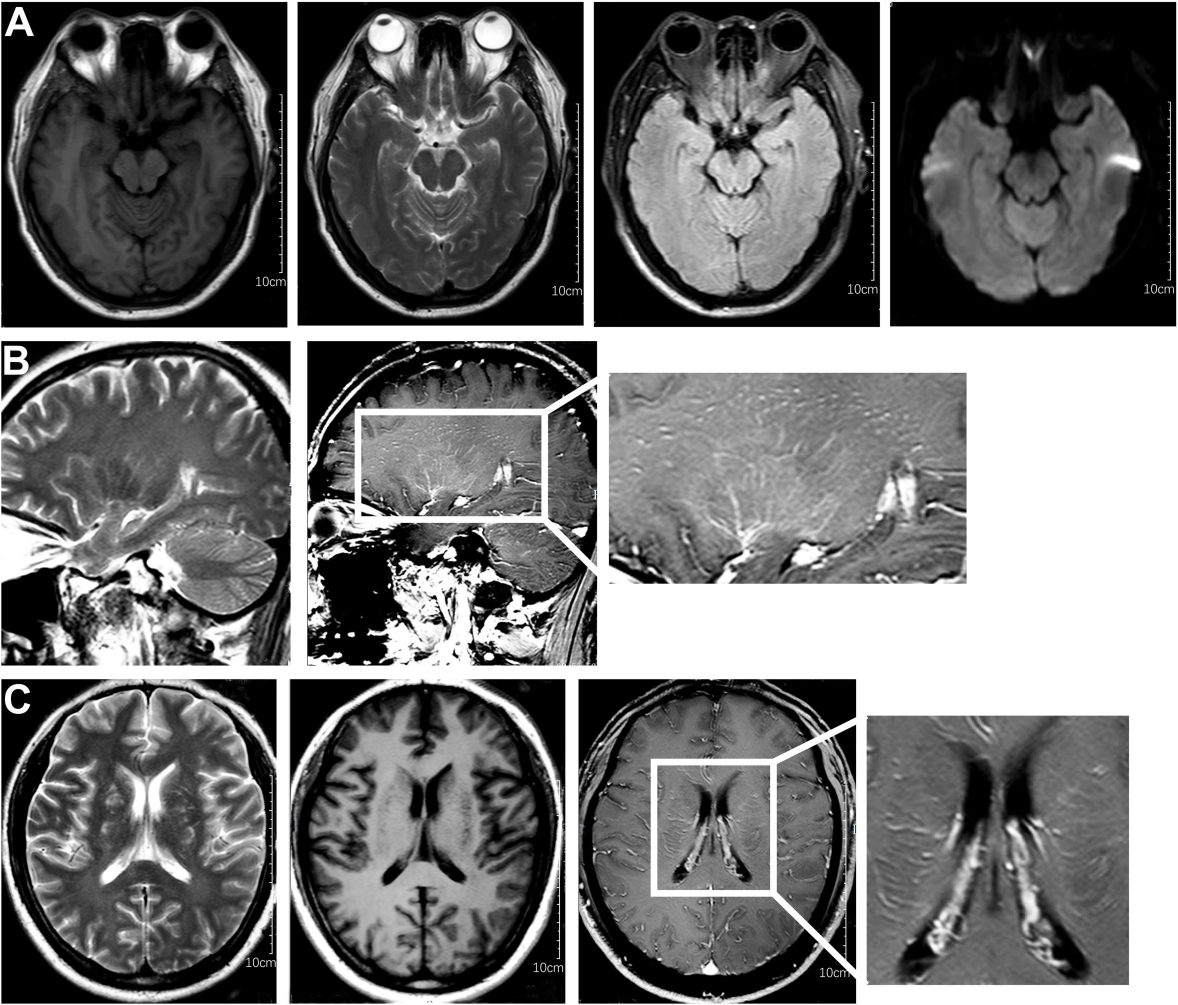
Characteristic brain MR images of autoimmune GFAP astrocytopathy. **(A)** Brain MRI showed the disappearance of T2/flair high signal in the cerebral cortex and bilateral hippocampus compared with previous brain MRI. **(B)** The sagittal section showed linear perivascular radial gadolinium enhancement in the white matter perpendicular to the ventricle. **(C)** Brain MR axial position suggests vascular enhancement perpendicular to the lateral ventricle.

After one week of antiviral treatment, the patient’s headache and fever resolved, and mental symptoms were reduced, but he still exhibited a gradually developing slow response and memory loss. In addition, abdominal distension and constipation occurred, and the patient experienced dizziness and discomfort when shifting from lying to sitting, indicating orthostatic hypotension. An abdominal CT scan revealed dilation of the small intestine with a gas-liquid plane. A repeat lumbar puncture was performed, which revealed an opening pressure of 150 mmHg, a protein content of 1.240 g/L, cerebrospinal fluid white cell count increased up to 304*10^6^/L (95% monocytes), and glucose of 3.11 mmol/L (**Table 1**). Tests for autoimmune encephalitis antibody panels were negative. Antibodies related to central nervous system demyelinating, including anti-aquaporin-4 (AQP4) antibody, anti-myelin-oligodendrocyte glycoprotein (MOG) antibody, and anti-myelin essential protein (MBP) antibody were negative. However, the patient obtained positive CSF test results for anti-glial fibrillary acidic protein (GFAP) antibodies, with titers of 1:1+ (**Figure 2**), confirmed with cell-based assay (CBA). Autoimmune screening of serum was negative for anti-nuclear antibodies (ANA), anti-extractable nuclear antigen (ENA), anti-DNA antibodies, anti-neutrophil cytoplasmic antibodies, and anti-phospholipid antibodies. A recheck of the brain MRI showed that the T2/FLAIR hyperintensity in the cerebral cortex and bilateral hippocampus had disappeared. A contrast-enhanced MRI revealed linear perivascular radial gadolinium enhancement in the white matter perpendicular to the ventricle (**Figure 3**).

**Figure 4:**
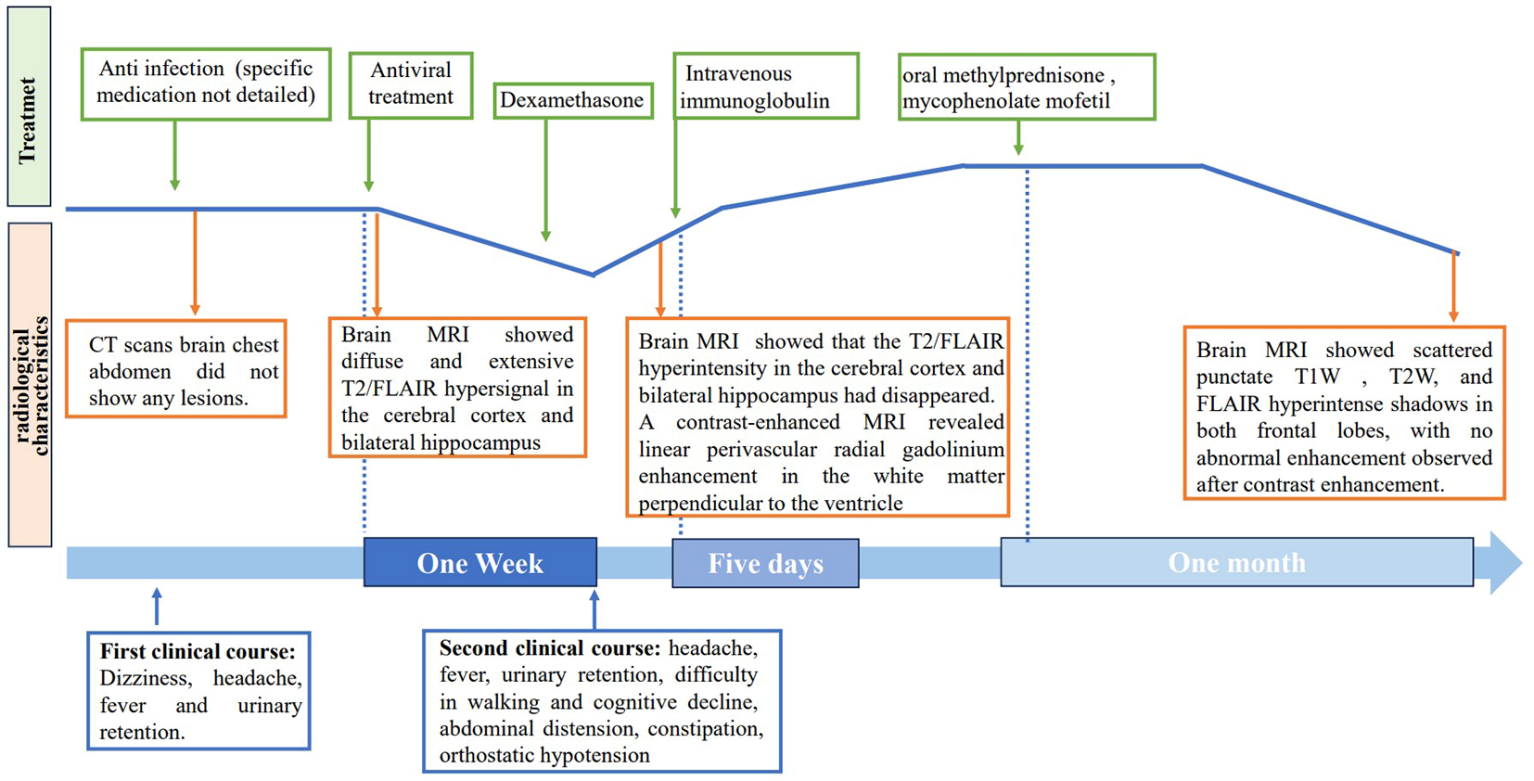
Timeline of clinical course. The clinical, radiological characteristics and treatment of the patient.

The patient was treated with a 5-day course of IVIG therapy (0.4 g/kg/d), which resulted in gradual clinical improvement. Scale evaluation using the Mini-Mental State Examination (MMSE) scored 15/30, and the Montreal Cognitive Assessment (MoCA) scored 15/30. The prognosis was evaluated using the modified Rankin Scale (MRS) and scored 2, indicating a good prognosis. In the later stage, oral methylprednisone was used sequentially, combined with the immunosuppressant mycophenolate mofetil. Following one month of oral medication, the patient returned to the hospital for a reexamination. Cognitive function, as assessed by the MMSE and MoCA improved, with scores of 21/30 and 23/30, respectively. The MRS scored 1. The CSF white blood cell count decreased to 45*10^6^/L (99% monocytes), protein levels decreased to 0.580 g/L, and glucose levels increased to 2.86 mmol/L (**Table 1**). Contrast-enhanced brain MRI showed reduced lesion enhancement. One month later, the patient underwent another CSF examination, which revealed an average white blood cell count of 11*10^6^/L (95% monocytes), decreased protein levels of 0.340 g/L, and glucose levels of 3.12 mmol/L (**Table 1**). Brain MRI showed scattered punctate T1W, T2W, and FLAIR hyperintense shadows in both frontal lobes, with no abnormal enhancement observed after contrast enhancement. Cognitive function improved, with MMSE and MoCA scores increasing to 29/30 and 30/30, respectively. The MRS score was 0, indicating a good prognosis. The patient responded well to treatment and continued to receive mycophenolate mofetil immunotherapy. Follow-up is ongoing. The clinical and radiological characteristics and treatment were summarized in **Figure 4**.

## Discussion

**Figure 5:**
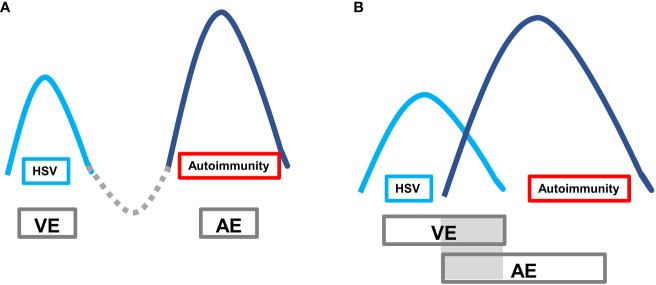
Autoimmune encephalitis secondary to viral encephalitis with typical bimodal encephalitis phenotype. The first peak is the VE period, and the second peak is the AE period. **(A)** Classic clinical phenotype: after the VE peak period treatment, it passes through the valley period of remission period, and then the AE peak period follows; **(B)** VE peak period overlaps with the AE peak period, lacking the valley period when the VE peak is generally not fully treated.

Autoimmune encephalitis (AE) caused by viruses, especially herpes simplex virus encephalitis (HSVE), is increasingly recognized as a significant health concern. The pathogenesis of VE-associated AE is thought to be related to immune responses triggered by the lysis of neurons and subsequent release of antigens following herpes simplex virus infection (2). The typical clinical presentation of VE-associated AE is a “bimodal encephalitis” phenotype characterized by two peak stages of the disease. The first peak stage is typically caused by virus invasion into the temporal lobe, frontal lobe, and limbic system. The second peak stage, secondary AE, is driven by pathogenic AE-related antibodies. However, a minority of cases may present with a unimodal or pseudounimodal phenotype due to self-limited mild symptoms during the AE stage. In some cases, there may be two peaks without a remission interval (referred to as a “valley stage”) when one peak has not been fully treated. During this time, there is concurrent brain inflammation caused by two mechanisms, with overlapping double peaks (**Figure 5**).

GFAP astrocytic disease is an autoimmune disorder of the nervous system with a low incidence rate, and its etiology, pathology, and mechanism are not yet fully understood. Further studies are needed to improve diagnosis and treatment. Here, we report a case of GFAP astrocytopathy caused by VE, which presented as bimodal encephalitis and was refractory to glucocorticoid therapy.

The patient had a history of oral and labial herpes. In the early course of the disease, the patient presented with an acute onset of fever, headache, cough, and other upper respiratory infection symptoms. As the disease progressed, the patient developed difficulty urinating, slow response, and indifferent emotions. The patient was treated with cephalosporin in a local hospital for anti-infection, but the treatment was ineffective. The diagnosis of viral infection was supported by clinical features (headache, fever), positive serological testing (positive for HSV-1 IgG and CMV IgG), cerebrospinal fluid analysis (consistent with viral infection based on changes in cerebrospinal fluid pressure, protein, cell, and glucose levels), MRI results (widespread high signals in the cerebral cortex), and preliminary efficacy of acyclovir treatment. However, after one week of standard anti-viral treatment, the patient’s headache and fever resolved, but the patient still had a slow response and memory loss, along with symptoms of autonomic nerve dysfunction (intractable hiccup, hyperhidrosis, postural hypotension, and sinus tachycardia). The treatment plan was then adjusted. The combination of antiviral therapy and low-dose dexamethasone was still ineffective, which was inconsistent with the diagnosis of VE. A further differential diagnosis was considered, and a lumbar puncture was performed again, revealing a positive result for GFAP-IgG in the CSF, while AQP4 and MOG antibodies were negative. The radiological findings characterized the disease as autoimmune GFAP astrocytopathy, with striking radial linear periventricular enhancement. The patient gradually improved after receiving IVIG. However, many issues still need to be considered in this case.

The diagnosis of VE was clear in this patient, and HSVE was considered to be highly probable, and the diagnostic basis deserves consideration. The patient first developed fever, headache and Mild cognitive impairment, followed by mental symptoms and walking difficulties gradually appeared. Considering viral encephalitis first based on the patient’s cerebrospinal fluid results (3). The patient’s serology revealed positive HSV-1 IgG, indicating prior exposure. HSV-1 is a virus belonging to human herpes virus (HHV) family, which is neurotropic and can remain latent in the ganglia after acute infection, periodically infecting the peripheral and central nervous systems. The etiology of herpes simplex encephalitis (HSE) remains unclear, with studies suggesting it may be due to the reactivation of latent virus rather than primary infection (4). The patient had a history of oral and labial herpes. Before the onset of fever and other symptoms this time, there was recurrent herpes labialis. The patient had mental and behavioral abnormalities, Brain MRI showed that the medial part of the temporal lobe was involved, which is a common site of herpesviral encephalitis. Meanwhile, serum HSV-IgG significantly increased (5). Therefore, the possibility of Herpes simplex virus infection should be considered first (6).

It is worth considering the mechanisms underlying the induction of autoimmune response following viral infection. Post-viral encephalitis autoimmune encephalitis (PVEAE) has an incidence rate of 27%. While there are numerous clinical studies on anti-N-methyl-D-aspartate receptor (NMDAR) encephalitis secondary to HSE, the incidence of other autoimmune encephalitis subtypes is relatively low (7). In recent years, three mechanisms have gained recognition: first, the molecular mimicry hypothesis, in which the viral protein sequence of HSV stimulates an immune response, and the produced antibody mistakenly reacts with antigenic determinants such as NMDAR (8); second, the site of HSV invasion, which is consistent with the high expression site of NMDAR and other receptors, leading to the exposure and modification of antigenic determinants such as NMDAR, making them targets of autoimmune response (9); and third, after HSV infection, T and B cells activate and secrete large amounts of inflammatory cytokines (10), leading to an immune response in the central nervous system through the blood-brain barrier (11).

Autoimmune GFAP astrocytopathy is a disorder that warrants further consideration due to its mechanism of brain injury as the second peak of PVEAE. GFAP, an intermediate filament protein, is expressed in cells and is a crucial component of intermediate products of mature astrocytes (12). GFAP functions not only as a biological marker of astrocytes but also contributes to various biological processes, including maintaining astrocyte morphology, the blood-brain barrier, synaptic plasticity, regulating cell proliferation, and transporting vesicles and lysosomes in astrocytes (13). As a result, the GFAP antibody itself may not induce pathological changes. However, it can serve as a marker of cytotoxic T cell-mediated immune inflammatory response in the pathogenesis of autoimmune GFAP astrocytopathy (14).

GFAP is present in astrocytes, and cell damage results in severe damage to the blood-brain barrier. Immune-mediated astrocyte dysfunction leads to cytokine release and the recruitment of inflammatory cells, activating T cell-mediated cytotoxic immune responses that further damage the nervous system. As a result, patients present with a high number of leukocytes (>5×10^6^/L) in their cerebrospinal fluid and significantly increased protein levels, sometimes exceeding 1g/L. Furthermore, GFAP is slightly expressed in Schwann cells, the glial cells that form the myelin sheath of peripheral nerves, causing peripheral nerve cell damage in roughly 5% of patients. Therefore, tissues expressing GFAP protein, such as peripheral nerve, autonomic preganglionic fibers, spinal cord, and brain tissue, may be damaged during the onset of autoimmune GFAP astrocytopathy (15).

In summary, the clinical presentation of autoimmune GFAP astrocytopathy is characterized by subacute onset meningitis, encephalitis, myelitis, or a combination of these syndromes, as well as symptoms related to autonomic nerve and peripheral nerve injury. The symptoms of autoimmune GFAP astrocytosis vary depending on the lesion’s location and scope, and there is no apparent specificity. While hormone therapy has been reported to result in better outcomes, the patient in question also demonstrated promising results with immunoglobulin treatment, possibly due to the immune-related pathogenesis of the disease or the presence of different types of the disease that respond differently to hormone therapy (16).

## Conclusions

In this case report, we present a case of bimodal encephalitis, which includes VE and Autoimmune GFAP astrocytopathy, with no precise interval between the two, posing a diagnostic challenge. Thus, it is crucial to monitor the patient’s condition after treating viral encephalitis and be vigilant in promptly detecting autoimmune encephalitis. Autoimmune GFAP astrocytopathy is a novel form of autoimmune encephalitis, and there is limited clinical experience in its management. Notably, our patient responded favorably to IVIG therapy, contrary to the previously reported treatment with steroids, suggesting distinct underlying pathophysiological mechanisms. Hence, the optimal management of this disease warrants further investigation.

## Data availability statement

The original contributions presented in the study are included in the article/supplementary material. Further inquiries can be directed to the corresponding author.

## Ethics statement

Written informed consent was obtained from the participant/patient(s) for the publication of this case report.

## Author contributions

PC: Writing – original draft, Writing – review & editing. WH: Writing – review & editing. MY: Writing – review & editing. ZC: Writing – review & editing. YG: Writing – review & editing. XZ: Writing – review & editing. WC: Writing – review & editing, Writing – original draft.
